# Study on the Mechanism of Enhanced Early-Age Properties of Steel Slag Cement Mortar Through Modified Nano-SiO_2_

**DOI:** 10.3390/ma19071338

**Published:** 2026-03-27

**Authors:** Ridong Fan, Baiyang Mao

**Affiliations:** 1 School of Environmental Science and Engineering, Donghua University, Shanghai 201620, China; 2 Shanghai Research Institute of Building Sciences Co., Ltd., Shanghai 200032, China; m19702658741@163.com

**Keywords:** KH550, nano-SiO_2_, steel slag, early-age performance, cement hydration

## Abstract

To enhance the early-age properties of steel slag cement mortar and promote the resource utilization of metallurgical solid waste, in this study, nano-SiO_2_ (KH-NS) was modified using a KH550 silane coupling agent. The hydration kinetics and microstructure evolution were systematically analyzed by means of a macroscopic performance test (setting time and compressive strength) and multi-scale microscopic characterization (characterized by Fourier Transform Infrared Spectroscopy, Scanning Electron Microscopy, X-ray Diffraction, Thermogravimetry-Differential Thermal Analysis, and isothermal calorimetry). The influence mechanism of its content on the early performance of the steel slag cement system was systematically studied. Research findings indicate that at a given dosage, increasing the proportion of KH-NS results in a shorter setting time for steel slag mortar. When the KH-NS dosage reaches 1.5%, the initial and final setting times of steel slag mortar decrease by 24.21% and 21.20%, respectively. The addition of KH-NS effectively enhances the compressive strength of mortar, with a particularly pronounced effect on early strength prior to 14 h of curing. At a KH-NS dosage of 1.5%, the onset of the accelerated phase of hydration heat release in steel slag cement mortar is advanced by 2.5 h. Mechanistic studies indicate that KH-NS accelerates cement hydration by promoting C_3_S dissolution and C-S-H gel nucleation through interactions between surface silanol groups (Si-OH) and amino groups (-NH_2_). Furthermore, KH-NS refines the pore structure via a micro-aggregate filling effect, reducing the number of harmful pores and improving the pore size distribution. KH-NS continuously consumes Ca(OH)_2_ through pozzolanic reactions to generate C-S-H, with its reactivity increasing with higher dosage. Research confirms that KH-NS significantly enhances the early strength and density of steel slag mortar, providing both theoretical justification and technical support for developing low-carbon building materials based on solid waste with high dosage.

## 1. Introduction

Steel slag, as a primary by-product of the iron and steel smelting industry, accounts for approximately 15–20% of crude steel output. China’s annual steel slag discharge exceeded 130 million tons in 2022, whilst its comprehensive utilization rate has persistently remained below 30% [1]. The accumulation of vast quantities of steel slag not only occupies land resources but also poses risks of heavy metal leaching, presenting a critical bottleneck constraining the green development of the steel industry [2]. From the perspective of material properties, steel slag is rich in mineral phases such as tricalcium silicate (C_3_S) and dicalcium silicate (C_2_S), which possess potential cementitious activity. With properties similar to silicate cement, it can serve as a mineral admixture to replace 30–50% of cement in concrete systems. This approach reduces hydration heat release by 20–35% [3] while facilitating the large-scale utilization of metallurgical solid waste. However, the high vitreous content and well-developed crystalline structure of steel slag result in significantly lower early-stage hydration activity compared to cement. When its dosage exceeds 20%, the 3 d compressive strength of concrete decreases by over 40% [4], severely limiting its application in early-strength engineering materials and presenting a core technical barrier to the large-scale resource utilization of steel slag.

To address the issue of delayed early strength development in steel slag cement systems, existing solutions primarily employ regulatory strategies such as blending with sulfoaluminate cement [5,6], steam curing [7], and reinforcement with nanomaterials [8]. Among these, the use of aluminate cement blends and steam curing presents drawbacks such as higher costs or limited application scenarios [9,10]. Nano-silica (NS), owing to its exceptionally high pozzolanic activity and nucleation effect, can effectively enhance matrix density by promoting C-S-H gel deposition [11]. Nazari et al. [12] demonstrated that the compressive strength of high-strength concrete peaks at an NS dosage of 4%. However, increasing the dosage to 5% restricts the pozzolanic reaction due to insufficient Ca(OH)_2_, while the agglomeration effect of NS intensifies, leading to a decline in concrete compressive strength. Li et al. [13] demonstrated that when the NS dosage reached 1%, the compressive strength of pavement concrete increased significantly. However, when the dosage increased to 3–5%, agglomeration occurred due to enhanced interparticle attraction among NS particles with high specific surface area, leading to a deterioration in mechanical properties. This confirmed that the NS reinforcement efficiency exhibits a negative correlation with its specific surface area. Wang et al. [14] demonstrated that a 1% NS dosage in ultra-high performance concrete achieves maximum compressive strength by increasing C-S-H nucleation sites and optimizing the interface transition zone (ITZ). However, dosages exceeding 1% diminish these mechanisms due to agglomeration effects, leading to strength reduction. This reveals the significant impact of material system variability on the optimal NS dosage. Xu et al. [15] elucidated the multiscale regulatory mechanism of NS on the ITZ. At the macroscale, NS enhances mechanical transmission by elevating the elastic modulus of the ITZ to 50–80% of the matrix. At the micro-scale, it accelerates the hydration reaction, promoting the densification of the ITZ structure at 3 d. These two processes synergistically drive a significant increase in compressive strength. The above research findings indicate that NS significantly enhances concrete density and mechanical properties through pozzolanic effects and microstructural filling. However, its efficiency enhancement is constrained by the threshold effect of dispersion levels—excessive addition (>3%) triggers irreversible agglomeration of nanoparticles due to van der Waals forces, leading to increased defect density within the interfacial transition zone (ITZ). This may even create stress concentration points, resulting in deterioration of mechanical properties [16]. It is worth noting that existing dispersion strategies (such as ultrasonic treatment and surfactant modification) can partially alleviate the agglomeration issue [17]. However, within the steel slag cement composite system, conventional modification methods struggle to coordinate the interfacial reactions between NS and the reactive components of steel slag (f-CaO, Fe_2_O_3_), while failing to adequately account for the dynamic effects of steel slag incorporation on the system’s liquid-phase alkalinity and Ca(OH)_2_ distribution.

Silane coupling agents, as a class of surface modification materials with both organic and inorganic functional groups, have been widely used in cement-based material systems to improve the interface compatibility between inorganic phases and organic phases, and optimize the dispersion of nanomaterials [18,19]. Current research on silane modification in steel slag-containing cementitious systems is relatively limited and has obvious research limitations: most existing studies focus on the surface modification of steel slag particles themselves by silane coupling agents (e.g., KH550, KH560), aiming to reduce the hydrophilicity of the steel slag surface, improve the bonding strength between steel slag and cement hydration products, and thus moderately enhance the mechanical properties of steel slag cementitious composites [20]. A small number of studies have also explored the simple physical blending of silane coupling agents with steel slag cement systems, which has a certain positive effect on improving the impermeability and chloride ion resistance of the matrix [21]. However, these studies only focus on the single modification of the steel slag phase or the simple compounding of silane with the system and cannot fundamentally solve the core problem of the low early hydration activity of steel slag caused by its high vitreous content and stable crystalline structure. Moreover, the enhancement effect of such modification methods on the early strength of steel slag cementitious materials is limited, which makes it difficult to meet the application requirements of early-strength engineering materials for steel slag-based cementitious systems.

In terms of the combination of silane modification and nanomaterial reinforcement, existing studies have confirmed that silane coupling agents can effectively improve the dispersion stability of nano-SiO_2_ in pure cement or fly ash/blast furnace slag composite systems through chemical grafting, and the grafted functional groups can further enhance the interface bonding between nanomaterials and cementitious matrix, thus maximising the nano-reinforcement effect [19,22]. However, there are almost no relevant research reports on the application of silane-modified nano-SiO_2_ in steel slag cement composite systems at present. The steel slag cement system has its own unique material characteristics: on the one hand, it contains f-CaO, Fe_2_O_3_ and other metal oxide components that are not abundant in pure cement or conventional mineral admixture systems, which may produce complex interfacial reactions with silane-modified nano-SiO_2_; on the other hand, the hydration process of steel slag will cause dynamic changes in the liquid-phase alkalinity and Ca(OH)_2_ distribution of the system, which will affect the dispersion state of nanomaterials and the play of pozzolanic activity. Therefore, the regulation effect and action mechanism of silane-modified nano-SiO_2_ on the early hydration kinetics and microstructural evolution of steel slag cement systems cannot be directly deduced from the research results of pure cement or other composite systems, and it is urgent to carry out targeted in-depth research.

Based on the above scientific research gaps, this study takes the steel slag cement composite system with 25% steel slag replacement rate as the research object, systematically investigates the effects of KH550-modified nano-SiO_2_ with different dosages on the setting time, early mechanical properties, pore structure, and hydration kinetics of steel slag cement mortar. Through a variety of macroscopic performance tests and multi-scale microscopic characterization methods, the synergistic enhancement mechanism of KH-NS on the early-age properties of steel slag cement mortar is elucidated from the three dimensions of molecular functional group interaction, hydration kinetics regulation, and microstructural evolution, and the optimal dosage of KH-NS in the steel slag cement system is determined. This study is expected to fill the research gap of silane-modified nanomaterials in steel slag cement composite systems, provide a new technical approach for activating the latent early hydration activity of steel slag, and lay a theoretical and technical foundation for the high-dosage and high-value utilization of steel slag in low-carbon building materials.

## 2. Methodology

### 2.1. Raw Materials

**Figure 1:**
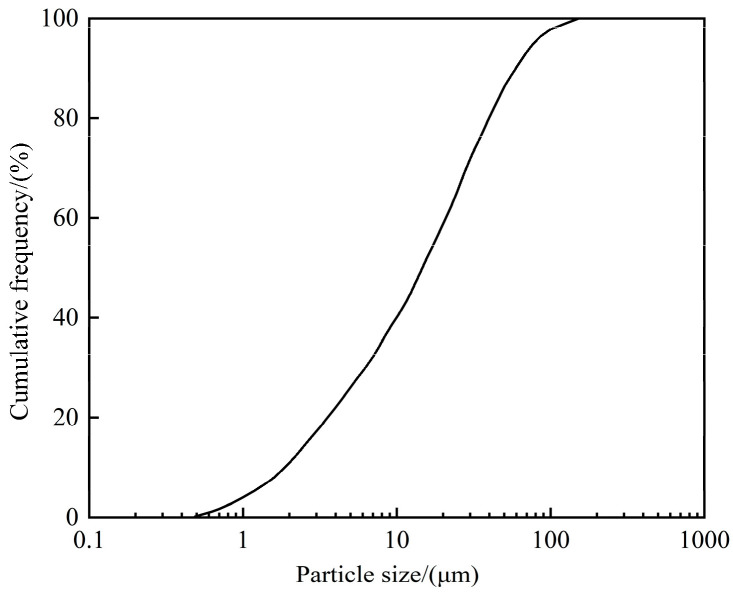
Particle size distribution of steel slag.

The cementitious material employed in this test was Conch P.O 42.5 ordinary Portland cement, with the chemical composition detailed in Table 1. Its bulk density is 3.15 g/cm^3^_._ The sand employed complies with ISO standards [23], whilst the steel slag utilized originates from an alkaline oxygen-converter steel slag produced by a certain steelworks. Steel slag is ground into powder with particle sizes smaller than 200 microns; its bulk density is 3.42 g/cm^3^. The particle size distribution is illustrated in Figure 1, with the chemical composition detailed in Table 2. NS with an average particle size of 15 nm and 99.5% purity was used. Tap water was employed for preparing the mortar. Silane coupling agent KH550, analytical grade, Shanghai Aladdin Biochemical Technology Co., Ltd. (Shanghai, China)

### 2.2. Preparation of KH-NS

Surface modification of NS was achieved via a silane coupling agent grafting method, with the specific steps as follows. Add 10.0 g of NS to 50 g of anhydrous ethanol. Process using an ultrasonic cell disruptor (power 600 W, frequency 20 kHz) for 1 h to obtain a suspension. Add drops of 0.1 mol/L formic acid solution to the suspension to adjust the system pH to 5.0 ± 0.2. Subsequently, slowly incorporate 5.0 g of KH550 silane coupling agent whilst maintaining magnetic stirring for 30 min to facilitate pre-hydrolysis. Transfer the mixture to a constant-temperature oil bath with a heat collector (Model DF-101S, Shanghai Yuezhong Instrument Equipment Co., Ltd., Shanghai, China) and reflux for 12 h at 100 ± 2 °C under mechanical stirring (100 r/min). Immediately after the reaction, vacuum filtration was performed (0.08 MPa). The product was washed three times sequentially with anhydrous ethanol and deionized water to remove unreacted coupling agent. Subsequently, the product was dried in a vacuum oven (70 °C) for 4 h to constant weight. Finally, it was cooled to room temperature in a desiccator, yielding KH550-modified nano-SiO_2_ (KH-NS).

The functional groups of KH-NS were characterized using a Nicolet 5DXC Fourier Transform Infrared Spectrometer (Thermo Nicolet, Madison, WI, USA) (FTIR, KBr pellet method, wavelength range 500 cm^−1^ to 4000 cm^−1^). The thermal stability of KH-NS was analyzed using a NETZSCH STA 2500 thermogravimetric analyzer (NETZSCH-Gerätebau GmbH, Selb, Germany). Testing was conducted in a nitrogen atmosphere within a temperature range of 0 °C to 900 °C, with a heating rate of 10 °C/min. The microstructure of KH-NS was observed using an FEI NOVA 400 field emission scanning electron microscope (FEI Company, Brno, Czech Republic) (SEM, gold sputter coating, acceleration voltage 15 kV).

### 2.3. Preparation of Mortar Specimens

In this study, the preparation of mortar samples strictly followed the ratio design and standardized operation process. A water–binder ratio of 0.5 was used as a fixed parameter, the composite cementitious system of 25% cement was replaced by steel slag, and different amounts of KH-NS modified nano-silica were added. The specific preparation steps were as follows. According to the test ratio, the raw materials were accurately weighed. The cementitious material was P.O 42.5 ordinary Portland cement and ground steel slag (mass ratio 3:1, steel slag replaced cement 25%), and the mass ratio of cementitious material to ISO standard sand was 1:3; the contents of KH-NS were 0% (control group), 0.5%, 1.0%, and 1.5% of the total mass of cementitious materials, respectively. The mixing water was tap water, and the dosage was determined according to the water–binder ratio of 0.5. The well-weighed KH-NS powder was mixed with all the mixing water and stirred at high speed for 5 min to obtain a uniform KH-NS water dispersion to avoid the agglomeration of nanoparticles in the subsequent mixing. The weighed cement and steel slag powder were placed in the mortar mixer for dry mixing treatment. The stirring speed was 300 r/min, and the dry mixing time was 2 min, so that the cementitious material was mixed evenly and the initial uniformity of the system components was ensured. The pre-dispersed KH-NS water dispersion was slowly poured into the mixer, and the speed was adjusted to 500 r/min and wet-mixed for 3 min, so that the cementitious material and the dispersion were fully fused to form a uniform cementitious slurry. The well-weighed ISO standard sand was added to the above cementitious slurry, the speed of the mixer was increased to 800 r/min, and the mixing was continued for 4 min. Finally, a uniform steel slag cement mortar mixture was prepared. The mixed mortar mixture was quickly poured into the mortar test mold. The test mold was placed on the vibration table and vibrated for 1 min to vibrate and compact. During the vibration process, the excess mortar on the surface of the test mold was scraped, and the surface of the test mold was smoothed. The molded test mold was placed in an environment with a temperature of 20 ± 2 °C and a relative humidity of ≥90% for 24 h. After the curing time was reached, the demolding operation was performed to ensure the integrity of the test block and avoid demolding damage. The mortar test block, after demolding, was immediately put into the standard curing room for curing. The curing conditions were a temperature of 24 ± 2 °C and relative humidity ≥98%, which were used for subsequent performance tests.

### 2.4. Testing Method

#### 2.4.1. Setting Time Test

The initial and final setting times of the cement mortar were determined using a Vicat apparatus (Wuhan Xigaohua Electric Co., Ltd., Wuhan, China) in accordance with the Chinese national standard GB/T 1346-2011 [24]. The test environment was maintained at a constant temperature of 20 ± 1 °C and a relative humidity (RH) of ≥90%.

#### 2.4.2. Mechanical Property Test

Specimens with dimensions of 40 mm × 40 mm × 160 mm were fabricated following GB/T 17671-2021 [23]. The specimens were cured in a standard curing chamber at 20 °C with RH ≥ 95% for curing ages of 6 h, 8 h, 10 h, 14 h, 18 h, 1 d, 3 d, 7 d, and 28 d, respectively. Flexural and compressive strengths were tested using a microcomputer-controlled universal testing machine (Jinan Huayue Testing Machine Co., Ltd., Jinan, China) at a loading rate of 2.4 kN/s. Each test group included three parallel specimens, and the average value was adopted as the final result.

#### 2.4.3. Porosity Test

The pore size distribution of specimens cured for 3 d was measured using an Auto Pore IV 9520 mercury intrusion porosimeter (MIP, Micromeritics Instrument Corporation, Norcross, GA, USA) with a pressure range of 1.5–350 kPa (low pressure) and 140–420 MPa (high pressure), based on the Washburn equation. Before testing, the samples were crushed into particles of 3–5 mm in size, immersed in isopropanol for 24 h to terminate hydration, and subsequently vacuum-dried at 60 °C to a constant weight.

#### 2.4.4. Hydration Kinetics Test

An eight-channel TAM AIR isothermal microcalorimeter (TA Instruments, New Castle, DE, USA) was employed to continuously monitor the hydration heat release rate for 3 d at a constant temperature of 20 ± 0.02 °C. The effect of KH-NS with different dosages on the hydration kinetics of the cementitious system was investigated. In order to eliminate the interference of inert aggregates and ensure the accuracy of the hydration heat measurement, the test was carried out on the cement slurry, and the water–binder ratio and KH-NS dosage were the same as those of the mortar sample.

#### 2.4.5. Microscopic Test

XRD patterns were recorded using a Bruker D8 ADVANCE X-ray diffractometer (Bruker AXS GmbH, Karlsruhe, Germany) with Cu-Kα radiation (λ = 0.15406 nm). The scanning range was set to 5°~65° with a step size of 0.02° and a scanning rate of 4°/min. Phase identification and analysis were performed using Jade 9.0 software. Thermogravimetric–differential thermal analysis (TG-DTA) was conducted using a NETZSCH STA 2500 simultaneous (NETZSCH Analyzing & Testing, Selb, Germany) thermal analyzer under a nitrogen atmosphere with a flow rate of 50 mL/min. The temperature was raised from room temperature to 1000 °C at a heating rate of 10 °C/min. The weight loss rate of hydration products and the characteristic phase transition temperatures were calculated from the TG-DTA curves. The mass of Ca(OH)_2_ was determined using Equation (1).

(1) $$m_{Ca{\left(OH\right)}_{2}}=m_{H_{2}O}\times \frac{M_{Ca{\left(OH\right)}_{2}}}{M_{H_{2}O}}$$

In the equation, *m*_*Ca*(*OH*)_2__ represents the mass of *Ca*(*OH*)_2_; *m*_*H*_2_*O*_ represents the weight loss of water (*H*_2_*O*) obtained from the TG curve; *M*_*Ca*(*OH*)_2__ is the molar mass of *Ca*(*OH*)_2_, taken as 74.09 g/mol; *M*_*H*_2_*O*_ refers to the molar mass of *H*_2_*O*, adopted as 18.02 g/mol.

## 3. Results and Discussion

### 3.1. Characterization of KH-NS

**Figure 2:**
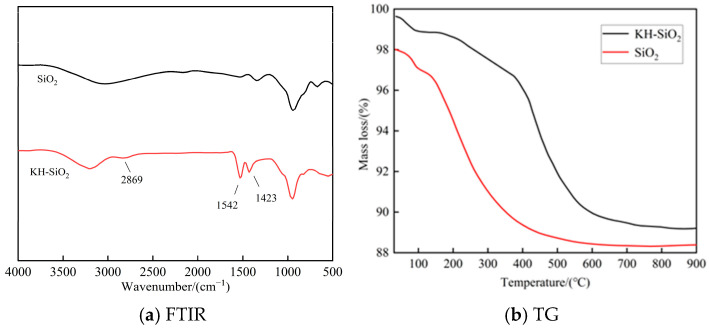
Characterization curve of KH-NS. (**a**) FTIR; (**b**) TG.

Figure 2a compares the FTIR spectral characteristics of pristine NS and KH-NS, clearly illustrating the evolution of surface functional groups on the nanoparticles before and after modification. The characteristic peak at 2869 cm^−1^ in the figure is attributed to the symmetric stretching vibration of the -CH_2_ group on the alkyl chain of the KH550 molecule [25]. This peak is absent in the original NS spectrum, and its appearance directly confirms that the organic chain segment of the silane coupling agent has been successfully grafted onto the surface of the NS particles through chemical action, thereby achieving the effective introduction of organic functional groups. Secondly, the characteristic peaks at 1542 cm^−1^ and 1423 cm^−1^ correspond respectively to the in-plane bending vibration of surface silanol groups (Si-OH) and the shear vibration of amino groups (-NH_2_) [26]. Compared to the original NS, the intensities of these two peaks exhibit regular variations, indicating that the Si-OH groups generated from the hydrolysis of KH550 molecules react with the native Si-OH groups on the surface of the NS particles via a condensation reaction. This forms stable Si-O-Si covalent bonds, thereby achieving chemical bonding between the coupling agent and the nanoparticles [27].

It is noteworthy that the synergistic appearance of the aforementioned characteristic peaks corresponds with a significant reduction in the half-width of the hydroxyl vibration peak at 3440 cm^−1^ observed in pristine NS. The hydroxyl peak at 3440 cm^−1^ primarily originates from the stretching vibration of surface silanol groups on NS. The reduction in its half-width indicates a more uniform distribution of surface silanol groups, alongside a decrease in the number of free hydroxyl groups due to condensation reactions [28]. This further corroborates at the molecular level that a chemical interaction occurs between KH550 and NS, rather than mere physical adsorption. Based on the combined spectral analysis, it is conclusively demonstrated that KH550 successfully modifies the surface of NS. Its core mechanism involves a dual chemical process comprising silanol condensation and the directed arrangement of amino groups. The silanol condensation reaction provides a chemical bonding foundation for the stable coupling of organic segments with nanoparticles, whilst the oriented arrangement of amino groups (-NH_2_) on the particle surface enables regulation of the electron cloud distribution via intermolecular forces [29]. This establishes the molecular structural basis for subsequently suppressing nanoparticle agglomeration and enhancing their dispersion within cement-based systems.

Figure 2b presents the TG curves before and after NS modification. This characterization provides thermodynamic evidence for the interaction between KH550 and NS from the perspectives of thermal stability and chemical composition evolution, serving as crucial complementary validation for the functional group analysis via FTIR. The TG curve of unmodified NS exhibits two characteristic weight loss stages, reflecting its inherent structural features and surface reactivity. The first stage concentrates on the 50–200 °C range, where weight loss stems from the desorption of physically adsorbed water and the condensation dehydration reaction between surface silanol groups (Si-OH). This process constitutes a reversible physicochemical transformation and represents a characteristic reaction of surface hydroxyl groups on nanoparticles [30]. The second stage occurs between 200 and 800 °C, corresponding to the progressive condensation of deep-seated silanol groups within the nanoparticles. This irreversible reaction reflects the strong interactions between hydroxyl groups within the particles, with the rate and magnitude of weight loss directly indicating the content of deep-seated hydroxyl groups [31]. The TG curve of KH-NS exhibits three characteristic weight loss stages, with the newly identified second weight loss stage (228–485 °C) serving as the core thermodynamic evidence for the successful grafting of KH550 [28]. The first stage (50–200 °C) remained consistent with the unmodified sample, still exhibiting desorption of physically adsorbed water and dehydration via surface silanol condensation, indicating that the modification did not alter the fundamental reaction characteristics of the NS surface. The weight loss observed in the second stage (228–485 °C) is primarily attributed to the thermal decomposition of the amino group (-NH_2_) within the KH550 molecule and the oxidative cracking of the organosiloxane chain segments. This temperature range closely corresponds to the characteristic decomposition temperature of the organic chain segments in silane coupling agents [27]. Furthermore, the proportion of weight loss exhibits a positive correlation with grafting density, directly demonstrating that KH550 and NS do not form a simple physical mixture but rather establish a stable bonded state. The third stage (485–800 °C) corresponds entirely to the second stage observed in the unmodified samples, representing a condensation reaction of deep-seated silanol groups. This indicates that the surface modification did not disrupt the internal structure of the NS, acting solely upon the particle surfaces.

Compared to unmodified NS, KH-NS exhibits a significantly increased total weight loss rate, with the newly observed weight loss phase demonstrating distinct temperature specificity. This excludes thermal behavior changes attributable to physical adsorption. Were this the case, KH550 molecules would readily desorb with moisture at lower temperatures (<200 °C), precluding the formation of an independent high-temperature weight loss phase [32]. Combined with evidence from the characteristic peak of Si-O-Si covalent bonds in FTIR, the three-stage weight loss phenomenon observed in the TG curve further confirms from a thermodynamic perspective that KH550 has successfully grafted onto the NS surface via chemical bonding, rather than through simple physical coating or adsorption. This stable chemical bonding structure provides the fundamental framework for subsequent KH-NS to withstand the corrosive effects of the hydration environment within cement-based systems and sustain its modifying action. Concurrently, the weight loss rate and magnitude during the second stage can indirectly quantify the grafting efficiency of KH550, offering thermodynamic evaluation criteria for optimizing the modification process parameters.

**Figure 3:**
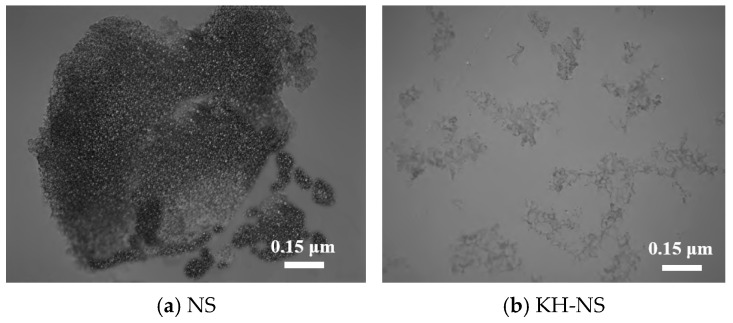
SEM images of NS before and after modification. (**a**) NS; (**b**) KH-NS.

Figure 3 provides a visual comparison of microstructural features before and after NS modification via scanning electron microscopy (SEM). This reveals the regulatory effect of KH550 modification on nanoparticle dispersion behavior at the mesoscale, establishing a microstructural foundation for understanding its role within the steel slag cement system. NS (Figure 3a) exhibits a typical three-dimensional reticular topography, with nanoparticles tightly aggregated through hydrogen bonding interactions formed by surface silanol groups (Si-OH), forming larger-sized clustered agglomerates. The interfaces between particles are indistinct and densely packed. The essence of this phenomenon lies in the fact that nanoparticles possess an extremely high specific surface area and surface energy, resulting in van der Waals forces between particles significantly exceeding the dispersive forces. Compounded by the hydrogen bonding interactions of silanol groups, this ultimately triggers a pronounced tendency towards agglomeration [33]. This agglomeration behavior impedes the uniform distribution of nanoparticles within cement-based systems, not only failing to fully realize their nano-reinforcement effects but also potentially acting as stress concentration points that degrade the mechanical properties of the matrix [31]. Compared to NS, the microstructure of KH-NS modified with KH550 (Figure 3b) exhibits a marked transformation. The originally dense, clustered agglomerates have been completely dissociated, forming discrete particles arranged in chains. Individual particles are clearly distinguishable, with significantly enhanced dispersion uniformity and spatial distribution rationality. The core mechanism underlying this morphological evolution lies not only in the fact that after KH550 is grafted onto the surface of NS via Si-O-Si covalent bonds, but its amino-containing organic long chains also form a “stereochemical barrier” on the particle surface. This barrier effectively prevents direct contact between adjacent nanoparticles, thereby weakening the strength of hydrogen bonding and van der Waals forces. Consequently, the formation and growth of agglomerates are suppressed [34].

### 3.2. Setting Time

**Figure 4:**
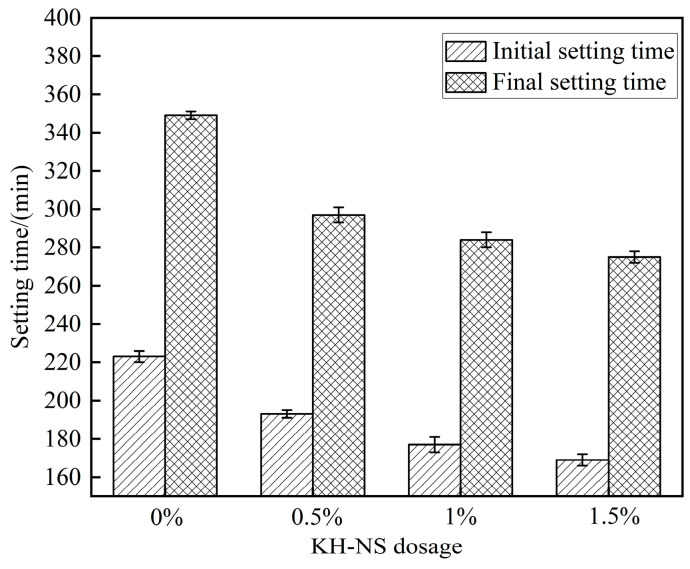
The setting time of steel slag cement mortar.

Figure 4 visually illustrates the regulatory patterns of setting time in steel slag cement mortar under varying KH-NS dosages. From a macro-performance perspective, this confirms the enhancing effect of KH-NS on the early hardening process of steel slag-based cementitious systems. It provides direct data support for addressing the core challenges of steel slag mortar, namely “delayed early setting and insufficient hydration activity”. Experimental data indicate that as the KH-NS admixture content progressively increased from 0% to 1.5%, the initial setting time of the mortar decreased continuously from 223 min in the control group to 169 min, representing a reduction of 24.21%. The final setting time decreased from 349 min to 275 min, representing a reduction of 21.20%. This result clearly demonstrates that within the admixture range defined in this study (0–1.5%), the KH-NS admixture content exhibits a significant negative correlation with mortar setting time. Higher admixture content accelerates the setting rate, with the most pronounced acceleration effect observed at a 1.5% admixture content. This reduction effectively compensates for the setting delay caused by slag admixture, thereby creating conditions for the application of slag mortar in early-strength projects.

The essence of this accelerated setting phenomenon lies in the multiple synergistic regulation of cement hydration by KH-NS. The specific mechanism of action can be elucidated from two aspects. Firstly, the silanol groups (Si-OH) and amino groups (-NH_2_) enriched on the KH-NS surface exhibit exceptionally high pozzolanic activity, and their synergistic interaction accelerates the hydration reaction [29]. On the one hand, the silanol group, as a highly reactive site, rapidly binds with Ca^2+^ ions in the cement hydration liquid phase, thereby reducing the liquid-phase Ca^2+^ concentration. This disrupts the dissolution–precipitation equilibrium of C_3_S, significantly promoting its dissolution and hydration [35]. On the other hand, the amino group (-NH_2_) readily undergoes protonation to form -NH_2_^+^. This further consumes liquid-phase Ca^2+^ through electrostatic adsorption, providing the driving force for the continuous dissolution of C_2_S [36]. Moreover, the higher the KH-NS dosage, the greater the number of active sites, leading to increased Ca^2+^ consumption rates and enhanced C_2_S dissolution efficiency. Consequently, the hydration reaction rate accelerates, and the setting time is correspondingly shortened (this mechanism will be further validated in Section 3.5 on hydration kinetics testing). Secondly, KH-NS acts as an efficient nucleation template during cement hydration. The nanoparticles themselves possess an exceptionally high specific surface area and surface energy. Following modification with KH550, their dispersibility is significantly enhanced (Figure 3), enabling uniform distribution within the cement paste. This provides numerous heterogeneous nucleation sites for the formation of hydration products, primarily C-S-H gel. Compared to the spontaneous nucleation of amorphous hydration products, the nucleation sites provided by KH-NS significantly reduce the nucleation barrier for C-S-H gel formation. This promotes rapid deposition of hydration products and crystalline growth, thereby accelerating the hardening process of mortar and shortening setting time.

In summary, KH-NS achieves efficient regulation of the setting time of steel slag cement mortar through a dual mechanism: accelerating ion reactions at active sites and promoting product deposition via nucleation templating. This regulatory effect intensifies with increasing dosage. This not only validates the efficacy of KH-NS surface modification but also corroborates the microscopic characterization findings from FTIR and SEM analyses (grafting of functional groups and improved dispersion) from a macroscopic performance perspective, thereby establishing a logical closed-loop relationship between microstructure and macroscopic properties.

### 3.3. Compressive Strength

**Figure 5:**
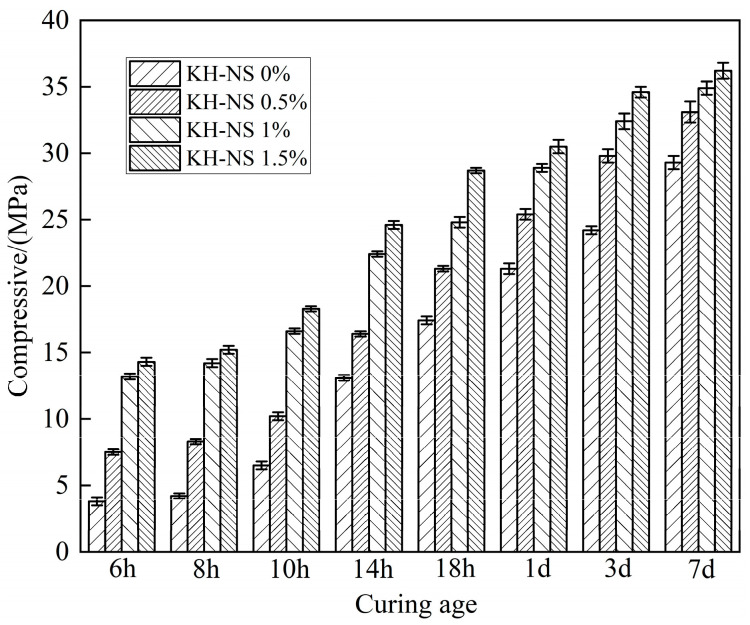
Compressive strength of steel slag cement mortar.

Figure 5 shows the compressive strength of steel slag cement mortar with different amounts of KH-NS. This provides direct mechanical evidence of KH-NS’s reinforcing effect on steel slag cement mortar, particularly in precisely addressing the core deficiency of “insufficient early strength” in steel slag mortars. Experimental data indicate a significant positive correlation between the dosage of KH-NS and the enhancement of mortar compressive strength. After 6 h of curing, the compressive strength of the 0.5%, 1%, and 1.5% admixture dosage groups increased by 97.4%, 247.4%, and 276.3%, respectively, compared to the control group. The 1.5% dosage group achieved nearly triple the early strength, highlighting KH-NS’s potent stimulation effect on early strength development. When the admixture dosage was fixed at 1.5%, the strength increase rates at 6, 8, 10, and 14 h reached 276.3%, 261.9%, 181.5%, and 87.8%, respectively, exhibiting a pattern of “rapid early gains followed by progressively narrowing increases with age”. At 28 d, strength remained 11.9% higher than the control group (control group: 39.4 MPa; modified group: 44.1 MPa), demonstrating that KH-NS not only significantly enhances early strength but also moderately improves long-term strength, achieving dual optimization of “early strength + sustained strength”.

The significant enhancement of early strength achieved by KH-NS is attributable, in part, to its accelerating effect on cement hydration and its nucleation-strengthening action. As previously described, the silicon hydroxyl (-Si-OH) and amino (-NH_2_) groups on the KH-NS surface rapidly consume liquid-phase Ca^2+^, promoting C_3_S dissolution. Simultaneously, they act as nucleation templates to lower the nucleation barrier for C-S-H gel formation, thereby accelerating the deposition of hydration products and mortar hardening [29,35]. For steel slag-based systems, the inherently low early-stage hydration activity of steel slag results in slow early strength development within the control group. The incorporation of KH-NS effectively compensates for this deficiency by externally stimulating the hydration process, thereby achieving a significant leap in early strength development prior to 14 h. On the other hand, this stems from the micro-aggregate filling effect of KH-NS and the long-term action of pozzolanic reactions. KH-NS exhibits excellent dispersibility, enabling it to function as a nano-scale micro-aggregate that fills internal pores within the mortar. This reduces the number of detrimental voids and enhances the system’s compactness, with a significant positive correlation observed between compactness and compressive strength. Moreover, KH-NS can continuously consume Ca(OH)_2_ through pozzolanic reactions to generate additional C-S-H gel throughout the entire hydration process. The C-S-H gel formed in later stages further fills pores and strengthens interfacial bonding, thereby achieving a steady increase in long-term strength [37].

It is noteworthy that KH-NS significantly enhances the early strength of steel slag mortar (maximum increase of 276.3% at 6 h), far surpassing the effects of conventional NS (where early strength improvements typically range from 50% to 150% at standard NS dosages of 1–3% [27,38]). This advantage stems from the KH550 modification, resolving the agglomeration issues inherent in conventional NS. The uniformly dispersed KH-NS fully exploits the nano-effect, preventing agglomerates from becoming stress concentration points and thereby maximizing its strengthening efficiency.

### 3.4. Pore Structure

**Figure 6:**
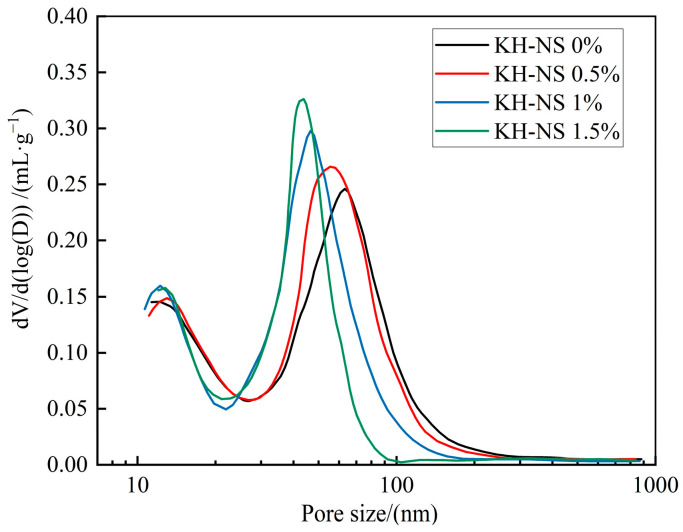
Pore distribution of steel slag cement mortar at 3 d.

Figure 6 illustrates the regulation patterns of pore structure in steel slag cement mortar after 3 d of curing at different KH-NS dosages (0%, 0.5%, 1%, 1.5%), revealing the core structural basis for KH-NS-reinforced mortar’s mechanical properties from the perspective of micro-compactness. Experimental data indicate that the total porosity of the control group reached 32.28%. Following the incorporation of 0.5%, 1%, and 1.5% KH-NS, the porosity decreased to 30.19%, 29.15%, and 28.21%, respectively, representing reductions of 6.5%, 9.7%, and 12.6%. This demonstrates a pronounced positive correlation where “higher dosage yields lower porosity”. More crucially, the optimization of the pore structure is manifested not only in a reduction in porosity but also in the directional restructuring of the pore size distribution. As the proportion of KH-NS increases, the pore structure within the mortar exhibits a distinct characteristic of “large pores migrating towards medium and small pores”. According to the classification standard for pores in cementitious materials (macropores > 50 nm, mesopores 10–50 nm, cementitious pores < 10 nm) [39], the proportion of detrimental macropores (which readily become stress concentration points and pathways for external erosion) in the reference group gradually diminishes. Conversely, the proportion of cementitious pores and mesopores, which enhance strength transfer and durability, significantly increases. This ultimately achieves a pore structure evolution towards a ‘more compact, more uniform, and higher-grade’ configuration. This pore size reconstruction effect is of significant importance, as the mechanical properties and durability of cementitious materials are governed not only by total porosity but predominantly by pore size distribution. The reduction of detrimental large pores directly mitigates the risk of stress concentration under loading while simultaneously inhibiting the ingress of external moisture and ions, thereby establishing a structural foundation for the long-term performance stability of mortar. One reason for this lies in the fact that the surface-enriched silanol groups (Si-OH) on KH-NS provide numerous heterogeneous nucleation sites for C-S-H gel formation [29]. This reduces the nucleation barrier for hydration products, facilitating the rapid deposition and growth of C-S-H gel within the macropores and on their walls, thereby achieving highly efficient filling of the macropores. On the other hand, KH-NS exhibits outstanding dispersibility, enabling it to act as a nanoscale micro-aggregate that directly fills the minute voids (particularly capillary pores) between cement hydration products. This refines pore size and reduces pore connectivity. This physical filling effect complements the chemical filling by hydration products, further enhancing the system’s density. It is worth emphasizing that pore structure optimization exhibits a highly significant positive correlation with compressive strength enhancement: the 1.5% KH-NS dosage group demonstrated the greatest reduction in porosity (12.6%), accompanied by the highest increase in 6 h compressive strength (276.3%). Conversely, the control group, characterized by elevated porosity and a high proportion of large pores, exhibited markedly lower early-stage strength. This correlation confirms the core mechanism by which KH-NS enhances mortar strength through optimization of the pore structure.

### 3.5. Hydration Kinetics

**Figure 7:**
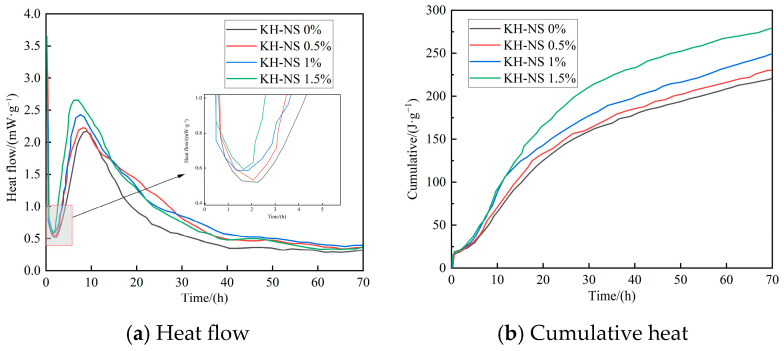
Hydration heat of steel slag cement mortar at 3 d. (**a**) Heat flow; (**b**) Cumulative heat.

Figure 7 reveals the regulatory patterns of different KH-NS dosages on the hydration heat release behavior of steel slag cement mortar. Figure 7a shows that the heat flow curves for all specimens exhibit five characteristic stages in the hydration exothermic process: an initial dissolution phase, an induction phase, an acceleration phase, a deceleration phase, and a stabilization phase. The incorporation of KH-NS does not alter the fundamental mechanism of the hydration reaction within the steel slag cement system; it merely accelerates the hydration process by regulating the reaction kinetic parameters. During the initial dissolution phase, the transient peak of high heat release originates from the rapid contact and dissolution reaction between cement and the active mineral phases in steel slag—such as tricalcium silicate (C_3_S) and dicalcium silicate (C_2_S)—with water, releasing substantial dissolution heat. Compared to the control group, the KH-NS-modified group exhibited a slight increase in the intensity of the initial exothermic peak, confirming its role in promoting the dissolution of mineral phases. Notably, as the KH-NS dosage increases, the onset time of the accelerated hydration phase is significantly advanced. The 1.5% dosage group exhibits a 2.5 h earlier onset compared to the control group. The core mechanism underlying this phenomenon can be further elucidated through microscopic characterization. On the one hand, the modified KH-NS exhibits excellent dispersibility (Figure 3), with its surface-enriched silanol groups (Si-OH) uniformly distributed throughout the slurry. This provides the C-S-H gel with a high density of heterogeneous nucleation sites, significantly lowering the nucleation barrier for hydration products. Consequently, the C-S-H gel rapidly precipitates during the acceleration phase, forming a continuous structure [29]. On the other hand, the amino group (-NH_2_) in the KH550 molecule readily undergoes protonation to form -NH_2_^+^, which efficiently captures Ca^2+^ ions in the hydrated liquid phase via electrostatic adsorption. This significantly reduces the liquid-phase Ca^2+^ concentration, thereby disrupting the dissolution–precipitation equilibrium of C_3_S/C_2_S [36]. Promoting the continuous dissolution of active mineral phases and the release of additional hydration ions provides a sufficient material basis for the vigorous reactions occurring during the acceleration phase.

Figure 7b shows the cumulative exothermic curve, which further quantifies the enhancement effect of KH-NS on the degree of hydration reaction. The total heat release within 3 d for the control group was 220 J/g, whereas the total heat release for the groups with 0.5%, 1%, and 1.5% KH-NS content increased to 230 J/g, 249 J/g, and 279 J/g, respectively. Compared to the control group, the cumulative heat release increased by 4.5%, 13.2%, and 26.8%, respectively, demonstrating a pronounced positive correlation where higher blending ratios yield greater cumulative heat release. This result demonstrates that KH-NS, through the dual pathways of “nucleation site optimization + ion adsorption regulation”, not only accelerates the rate of hydration reactions but also significantly enhances the overall degree of hydration. For the steel slag cement system, the high glass phase content and stable crystalline structure of steel slag result in low early-stage hydration activity, leading to a comparatively low cumulative heat release in the reference group. However, the addition of KH-NS precisely stimulates the latent cementitious activity of steel slag, promoting synergistic reactions with cement hydration products and thereby increasing the total heat of hydration. Moreover, the 26.8% increase in heat release in the 1.5% dosage group strongly correlates with the 276.3% rise in early strength (Figure 5) and the 12.6% decrease in porosity (Figure 6) observed in this group. This confirms the logical closed loop that “enhanced hydration promotes increased hydration products, resulting in a denser structure and ultimately leading to improved mechanical properties,” further corroborating the inherent consistency of KH-NS’s modification mechanism for steel slag-based cementitious systems.

### 3.6. Cement Hydration

**Figure 8:**
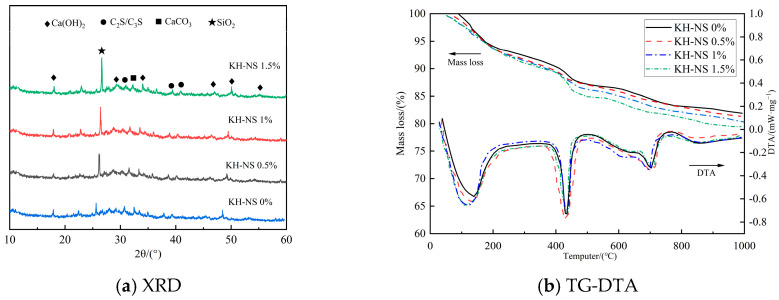
XRD and TG-DTA of steel slag cement mortar at 3 d. (**a**) XRD; (**b**) TG-DTA.

Figure 8a shows the XRD patterns of mortar at 3 days of age for different KH-NS dosages. Pattern analysis indicates that the primary phases in all specimens are calcium hydroxide (Ca(OH)_2_), silicon dioxide (SiO_2_), dicalcium silicate (C_2_S), tricalcium silicate (C_3_S), and calcium carbonate (CaCO_3_). No new characteristic diffraction peaks were observed. The incorporation of KH-NS does not alter the fundamental pathway of the hydration reaction within the steel slag cement system; it merely enhances the hydration effect by accelerating the existing reaction process. It is noteworthy that the peak intensity exhibits a pronounced regular variation. As the KH-NS dosage increases, the intensity of the SiO_2_ characteristic peak continues to rise. This phenomenon stems from a dual contribution. On the one hand, this stems from the direct signal superposition of unreacted NS particles within the KH-NS. On the other hand, more crucially, the cumulative effect of Si-O bonds within the amorphous C-S-H gel formed during the hydration reaction (though lacking sharp diffraction peaks, the C-S-H gel enhances the superimposed SiO_2_ diffraction signals in the system) indirectly corroborates the increase in total hydration products. Concurrently, the characteristic peak intensities of Ca(OH)_2_, C_3_S, and C_2_S progressively diminished. The weakening of the C_3_S/C_2_S peak intensity indicated an accelerated dissolution and consumption rate of the active mineral phases, whilst the reduction in Ca(OH)_2_ peak intensity was entirely consistent with the subsequent decrease in Ca(OH)_2_ content observed in the TG analysis [37]. The aforementioned phase evolution patterns conclusively demonstrate that the higher the KH-NS dosage, the more pronounced the effect on promoting cement hydration.

**Figure 9:**
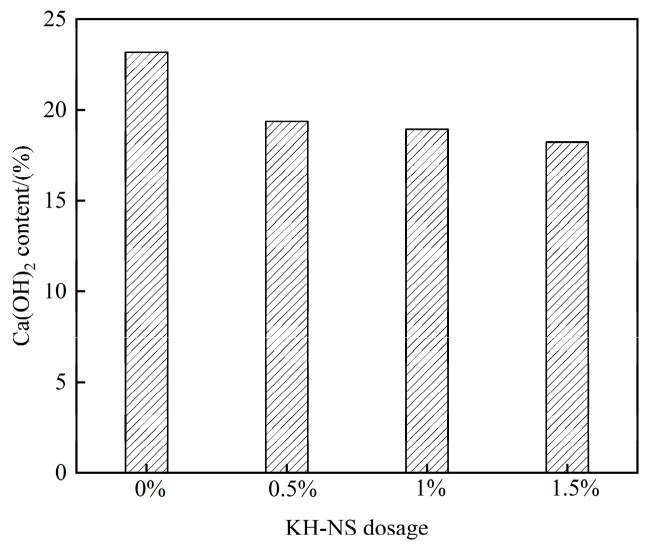
Ca(OH)_2_ content of steel slag cement mortar at 3 d.

Figure 8b shows the TG-DTA curve for the corresponding sample, exhibiting three distinct weight loss stages within the temperature range from room temperature to 1000 °C. The thermal behavior in each stage correlates unequivocally with specific phase transitions. The weight loss in the first stage (50–200 °C) stems from the de-bonding of hydration water between aluminate hydrates (AFt) and C-S-H gel [40]. The weight loss rate in this stage increases slightly as the KH-NS dosage rises. This correlates with the pattern of “increased hydration products” revealed by XRD analysis: the higher the C-S-H gel content, the greater the total amount of desorbed water. The sharp weight loss peak in the second stage (400–500 °C) is characteristic of Ca(OH)_2_ decomposing into CaO and H_2_O [41]. The intensity of this peak diminishes significantly with increasing KH-NS dosage, directly reflecting the reduction in Ca(OH)_2_ content. The weight loss peak observed in the third stage (600–750 °C) is attributed to the decomposition of different crystalline forms of CaCO_3_ (primarily formed by the reaction of Ca(OH)_2_ with atmospheric CO_2_) [42]. The variation in its intensity correlates with the decreasing trend in Ca(OH)_2_ content, further corroborating alterations in the hydration process. The Ca(OH)_2_ content at 3 d calculated using Equation (1) is shown in Figure 9: the control group exhibited a Ca(OH)_2_ content of 23.19%, while the content decreased to 19.39%, 18.93%, and 18.22%, respectively, after incorporating 0.5%, 1%, and 1.5% KH-NS. This represents a reduction of 16.30–21.43% compared to the control group, with the decrease becoming more pronounced as the KH-NS dosage increased.

This mechanism clearly demonstrates that KH-NS continuously consumes Ca(OH)_2_ through pozzolanic reactions, generating additional amorphous C-S-H gel. Within the steel slag cement system, Ca(OH)_2_ is a by-product of hydration reactions; its accumulation inhibits the further dissolution of C_3_S/C_2_S. The pozzolanic reaction of KH-NS consumes the excess Ca(OH)_2_, thereby disrupting this inhibitory equilibrium. Simultaneously, the generated C-S-H gel fills the pores and strengthens the structure [40]. The decreasing trend in Ca(OH)_2_ content observed in TG analysis aligns closely with the diminished intensity of characteristic Ca(OH)_2_ peaks in XRD spectra. This cross-validates KH-NS’s promotion of early hydration from both quantitative content and qualitative phase perspectives. Moreover, this enhancing effect intensifies with increasing dosage, perfectly aligning with the earlier observation that “higher dosages yield more pronounced strength gains and lower porosity”.

### 3.7. Mechanism Analysis

Based on the comprehensive test results of macroscopic performance (setting time, compressive strength), multi-scale microscopic characterization (FTIR, SEM, XRD, TG-DTA), and hydration kinetics (isothermal calorimetry, MIP), the enhancement effect of KH550-modified nano-SiO_2_ (KH-NS) on the early-age properties of steel slag cement mortar is not a single physical or chemical action, but a multi-scale synergistic enhancement effect formed by the coupling of “surface modification optimization–hydration reaction acceleration–micro-structure densification”. This effect constructs a complete logical chain from molecular-level functional group regulation to meso-level pore structure optimization, and finally to macro-level performance improvement, which fundamentally solves the technical bottleneck of low early hydration activity and poor early strength of steel slag cementitious systems. The specific action mechanism is elaborated from three core aspects, as follows.

#### 3.7.1. Dispersion Optimization and Functional Group Activation of KH-NS

The surface modification of raw NS by KH550 silane coupling agent is the fundamental prerequisite for realizing the nano-reinforcement effect in the steel slag cement system, which solves the two key problems of easy agglomeration and low active site exposure of raw NS. FTIR and TG characterization results show that KH550 is grafted on the surface of NS through Si-O-Si covalent bond formation (instead of simple physical adsorption) after pre-hydrolysis: the silanol groups (Si-OH) generated by KH550 hydrolysis undergo condensation reaction with the intrinsic Si-OH on the NS surface, and the organic chain segment with amino group (-NH_2_) is stably anchored on the NS surface. This chemical bonding structure forms a significant steric hindrance effect on the NS surface, which weakens the van der Waals force and hydrogen bonding between nanoparticles, making the originally dense agglomerated raw NS dissociate into discrete chain-like dispersed particles, and realizing the uniform distribution of KH-NS in the cementitious slurry.

Meanwhile, the successful grafting of KH550 makes the KH-NS surface expose dual active functional groups (Si-OH and -NH_2_) at the same time, which realizes the activation of the surface reactivity of nanoparticles: the Si-OH group provides a large number of heterogeneous nucleation sites for the formation of hydration products (C-S-H gel) in the subsequent hydration process, and the -NH_2_ group can participate in the regulation of ion concentration in the hydration liquid phase through protonation reaction. The synergistic exposure of the two functional groups lays a solid molecular foundation for the subsequent acceleration of the hydration reaction and the improvement of interfacial bonding strength between nanoparticles and cementitious matrix, and is the core guarantee for KH-NS to exert the nano-enhancement effect in the steel slag cement system with high alkalinity and complex ion environment.

#### 3.7.2. Dual-Path Acceleration of Hydration Reaction

KH-NS takes the activated surface functional groups as the core and accelerates the hydration reaction process of the steel slag cement composite system through the synergistic action of “nucleation template effect” and “ion regulation effect”, which fundamentally solves the problem of low early hydration activity of steel slag caused by high vitreous content and stable crystal structure, and becomes the core driving force for the shortening of mortar setting time and the significant improvement of early strength. On the one hand, the uniformly dispersed KH-NS takes the surface Si-OH groups as heterogeneous nucleation templates for C-S-H gel. The high specific surface area and surface energy of nanoparticles significantly reduce the nucleation barrier of C-S-H gel formation, making the hydration products deposit and crystallize rapidly on the KH-NS surface in the early stage of hydration. This effect shortens the induction period of the hydration reaction of the steel slag cement system and makes the onset time of the hydration heat release acceleration phase advance by 2.5 h when the KH-NS dosage is 1.5% (Figure 7a). XRD test results also show that the signal intensity related to SiO_2_ in the mortar increases with the increase of KH-NS dosage, which indirectly verifies the increase of C-S-H gel content formed by early hydration, and lays a material foundation for the early hardening and strength development of mortar.

On the other hand, the -NH_2_ groups on the KH-NS surface are prone to protonation in the alkaline hydration liquid phase to form -NH_3_^+^, which can efficiently capture Ca^2+^ ions in the liquid phase through electrostatic adsorption and reduce the concentration of Ca^2+^ in the hydration liquid phase. This ion regulation effect breaks the dissolution-precipitation equilibrium of tricalcium silicate (C_3_S) and dicalcium silicate (C_2_S) in cement and steel slag, promotes the continuous dissolution of these active mineral phases, and releases more hydration ions to participate in the formation of C-S-H gel and Ca(OH)_2_. At the same time, KH-NS continuously consumes the generated Ca(OH)_2_ through a pozzolanic reaction to generate additional amorphous C-S-H gel, which not only eliminates the inhibitory effect of Ca(OH)_2_ accumulation on the further hydration of C_3_S/C_2_S but also supplements the cementitious hydration products. TG-DTA test results show that the Ca(OH)_2_ content of mortar at 3 d is reduced by 16.30%~21.43% with the increase of KH-NS dosage (Figure 9), which fully verifies the continuous consumption of Ca(OH)_2_ by KH-NS. The dual-path hydration acceleration effect makes the cumulative hydration heat release of the system at 3 d increase by 26.8% when the KH-NS dosage is 1.5% (Figure 7b), and the hydration reaction degree of the steel slag cement system is significantly improved, which directly leads to the shortening of mortar setting time (24.21% reduction of initial setting time, 21.20% reduction of final setting time) and the explosive growth of early compressive strength (276.3% increase of 6 h strength).

#### 3.7.3. Microstructure Densification via Dual Filling Effect

The acceleration of the hydration reaction and the increase of hydration products further realize the densification of mortar microstructure through the dual filling effect of physical micro-aggregate filling and chemical hydration product filling of KH-NS, which is the key bridge connecting the micro-level hydration regulation and the macro-level mechanical property improvement of mortar. This effect optimizes the pore structure of mortar from both physical and chemical aspects, reduces the number of harmful pores, and improves the compactness of the cementitious matrix, thus significantly improving the compressive strength and structural stability of mortar. On the one hand, KH-NS, as a nano-scale micro-aggregate after uniform dispersion, can directly fill the capillary pores and macropores formed in the early hydration process of the steel slag cement system, realizing the physical optimization of the pore structure. MIP test results show that with the increase of KH-NS dosage, the total porosity of mortar at 3 d age is reduced from 32.28% (control group) to 28.21% (1.5% KH-NS group), a decrease of 12.6%, and the pore size distribution shows a significant characteristic of “large pores migrating to medium and small pores” (Figure 6). The reduction of total porosity and the decrease of harmful macropore content effectively reduce the stress concentration in the mortar matrix under load and improve the stress transfer efficiency of the cementitious system.

On the other hand, the C-S-H gel generated by the KH-NS accelerated the hydration reaction and the secondary C-S-H gel formed by the pozzolanic reaction realize the chemical filling of the pore structure: these hydration products are deposited on the wall of the original pores and the interface of cement/steel slag particles, filling the micro-pores and bridging the micro-cracks in the matrix, further optimizing the pore size distribution and improving the interfacial bonding strength between the aggregate and the cementitious paste. The combination of physical filling and chemical filling makes the mortar microstructure change from “loose and porous” to “dense and uniform”, which not only ensures the significant improvement of the early compressive strength of mortar, but also maintains the steady growth of long-term strength (11.9% increase of 28 d strength in the 1.5% KH-NS group). This dual filling effect is the direct reason for the improvement of mortar strength at all ages and also lays a structural foundation for the improvement of the long-term durability of the steel slag cement system.

In summary, KH-NS realizes the comprehensive enhancement of the early-age properties of steel slag cement mortar through the multi-scale synergistic mechanism of “dispersion optimization and functional group activation–dual-path hydration acceleration–dual filling microstructure densification”. This mechanism not only reveals the intrinsic law of the interaction between silane-modified nano-SiO_2_ and the steel slag cement system but also provides a theoretical basis for the high-value utilization of metallurgical solid waste (steel slag) in low-carbon building materials and lays a technical foundation for the development of high-performance cementitious materials with a high steel slag replacement rate.

## 4. Conclusions

This study systematically investigated the mechanism by which KH-NS regulates the early-age properties of steel slag cement mortar. The principal conclusions are as follows:

(1) As the dosage of KH-NS increases, the setting time of the mortar is uniformly shortened. Moreover, at a given dosage level, the greater the amount of KH-NS added, the shorter the setting time of the steel slag mortar mixture becomes. When the KH-NS admixture content was 1.5%, the initial setting time of steel slag mortar progressively decreased from 223 min to 169 min (a reduction of 24.21%), while the final setting time decreased from 349 min to 275 min (a reduction of 21.20%).
(2) The addition of KH-NS effectively enhances the compressive strength of steel slag mortar, with a particularly pronounced effect on early strength before 14 h of curing. At a KH-NS dosage of 1.5%, the 6 h strength increased by 276.3%, while the 28 d strength stabilized at 44.1 MPa (compared to 39.4 MPa for the control group), representing an 11.9% strength gain. Concurrently, at a KH-NS dosage of 1.5%, the mortar’s total porosity decreased from 32.28% to 28.21% (a reduction of 12.6%), with the pore size distribution shifting towards medium and small pores.
(3) With increasing KH-NS dosage, the onset time of the accelerated phase of steel slag mortar hydration heat release was brought forward by 2.5 h (in the 1.5% dosage group), with total heat release within 3 d increasing by 26.8%. The addition of KH-NS does not induce the formation of new hydration products in mortar and is capable of accelerating the cement hydration process. KH-NS can consume Ca(OH)_2_ through pozzolanic reactions to form C-S-H gel, with its reactivity increasing with higher dosage.
(4) KH-NS simultaneously maximizes the nano-effect through dispersion optimization and activates the latent cementitious activity of steel slag via functional group regulation. This approach ultimately enhances material performance while promoting the high-value utilization of metallurgical solid waste, thereby providing theoretical support for the accelerated development of low-carbon building materials.
(5) This study primarily focuses on the grafting configuration of the KH550 molecule on nanoparticle surfaces and its atomic-level interactions with the interface of the hydration products, but lacks verification through simulations based on first-principles calculations or molecular dynamics. Moreover, the tests primarily evaluated mortar performance under standard curing conditions. Systematic investigation remains necessary to determine the long-term durability and damage evolution patterns of KH-NS-modified slag mortar under complex and severe service conditions, such as sulfate attack, freeze–thaw cycles, and high temperatures.

## Figures and Tables

**Table 1:** Chemical composition of cement.

Composition	SiO_2_	Al_2_O_3_	Fe_2_O_3_	CaO	SO_3_	MgO	LOI
Mass fraction/%	22.29	6.80	4.63	58.23	2.42	0.97	4.66

**Table 2:** Chemical composition of steel slag.

Composition	CaO	Fe_2_O_3_	SiO_2_	CO_2_	MnO	MgO	Al_2_O_3_	TiO_2_	Other
Mass fraction/%	41.41	19.72	13.81	11.34	5.50	3.85	1.80	1.31	1.26

## Data Availability

The original contributions presented in this study are included in the article. Further inquiries can be directed to the corresponding author.
